# Supplementary material for: Predatory Odor Exposure as a Potential Paradigm for Studying Emotional Modulation of Memory Consolidation—The Role of the Noradrenergic Transmission in the Basolateral Amygdala
Source: Int J Mol Sci. 2024 Jun 14;25(12):6576. doi: 10.3390/ijms25126576 (PMC11204360; doi:10.3390/ijms25126576)

Supplementary materials Western blot gels

Gel 1-2

Total protein gels for C-veh, POE-veh and POE-prop during the Sample phase and the Test phase.

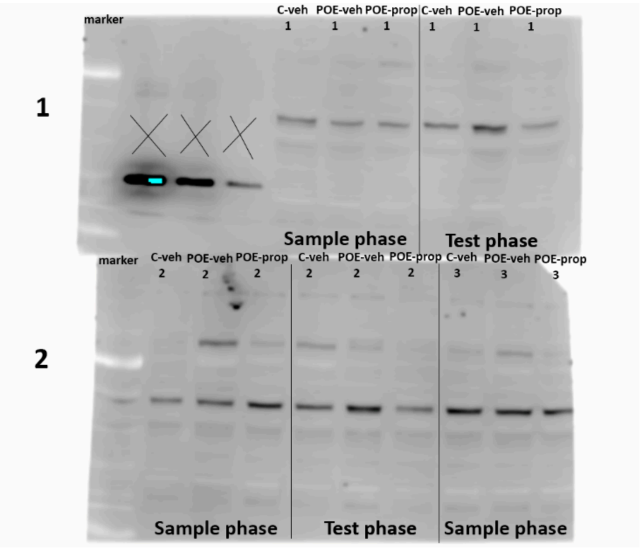

Gel 3-4

Total protein gels for C-veh, POE-veh and POE-prop during the Sample phase and the Test phase.

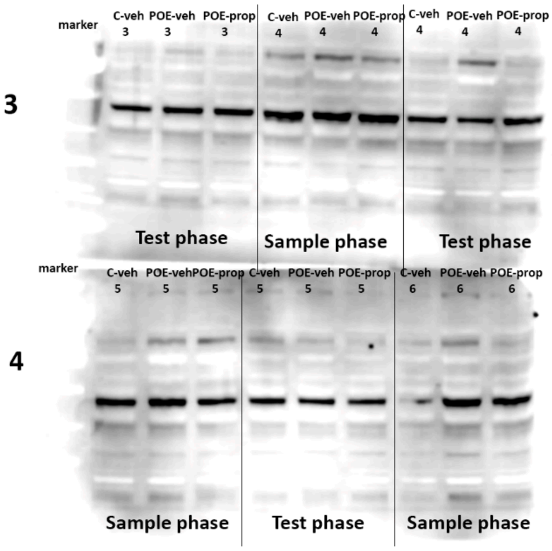

Supplement: Supplementary file 1 [file ijms-25-06576-s001.zip › Supplementary File S1.pdf]
