# Supplementary material for: Predatory Odor Exposure as a Potential Paradigm for Studying Emotional Modulation of Memory Consolidation—The Role of the Noradrenergic Transmission in the Basolateral Amygdala
Source: Int J Mol Sci. 2024 Jun 14;25(12):6576. doi: 10.3390/ijms25126576 (PMC11204360; doi:10.3390/ijms25126576)

Coronal brain sections of the Wistar rat brain. (A) Schematic drawings of the coronal sections showing the coordinates of the basolateral amygdala (BLA) according to the atlas of Paxinos and Watson (Paxinos and Watson, 2004). (B) Representative coronal brain sections representing the cannula and the injection tip placement (lower arrow).

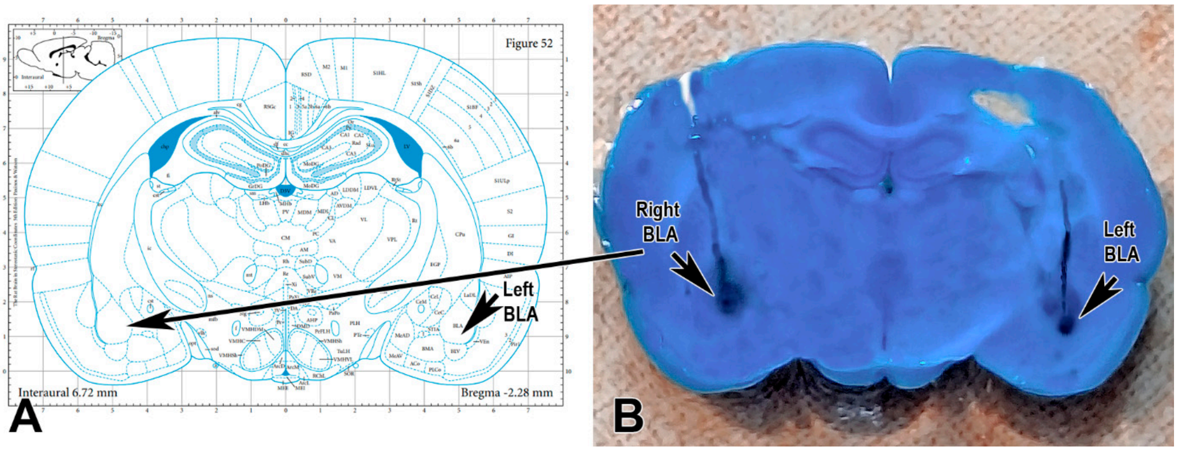

Supplement: Supplementary file 1 [file ijms-25-06576-s001.zip › Supplementary File S2.pdf]
